# Quality of Life in Hemophilia Complicated by Inhibitors

**Published:** 2012-04-01

**Authors:** P Bastani, K Pourmohamadi, M Karimi

**Affiliations:** 1 Student of Health Services Management, Tehran University of Medical Sciences, Tehran, Iran; 2 Health Services Management, Shiraz University of Medical Sciences, Shiraz, Iran; 3 Hematology Research Center, Shiraz University of Medical Sciences, Shiraz, Iran

**Keywords:** Quality of life, Hemophilia, Inhibitors, Iran

Dear Editor,

Inhibitors in patients with hemophilia are a rare complication which leads to pain, disability and impairment in quality of life. Recent advances in treatment have improved this situation, doubtful of whether it improves patients` quality of life or not.[1]

The overall prevalence of inhibitors in patients with hemophilia A or B in Iran is estimated to be approximately 9% and 3% respectively; patients with moderately severe (factor levels 1-5%) and severe hemophilia A (<1%) have a greater risk of developing inhibitors, with reported the prevalence between 7% and 20%.[2][3][4] The introduction and availability of bypassing products in treatment have definitively contributed to prolong patients` life expectancy and to improve their quality of life.[5] The management of hemophiliacs with inhibitors is particularly expensive in comparison with the treatment of hemophiliacs without inhibitors. The aim of this study was to evaluate the quality of life in severe hemophilia patients with inhibitor compared with quality of life of 52 non-inhibitor severe hemophilia patients.

This cross-sectional study was carried out in Shiraz Hemophilia Center, a major referral center in south of Iran. To reach the objectives of the study, the short form 36 questionnaire was applied. The informed consent was obtained before the study. Six severe hemophilia A patients with the age range of 11-32 years old, (<1% arriving) and high responders, high titer (>5 Bethesda uni) were selected. SF-36 was provided for patients retrospectively. The control group consisted of 52 non-inhibitor severe hemophilia A patients. To evaluate quality of life, both groups were interviewed by structured questionnaire SF-36.

The SF-306 questionnaire is considered as a standard instrument for patient-based health care outcome assessment.[6][7] It has been recently validated in Iran, and has been used in several studies ranging from epidemiological to clinical trials. Applicable to adults and adolescents, SF-36 assesses eight dimensions of Health related quality of life (HRQOL) which is related to the physical and mental components of health perception: physical functioning, rolephysical and bodily pain were more related to the physical component; social functioning, role, emotional and mental health were more related to the mental component; finally, energy/vitality and general health relate to both components. These 8 dimensions can be classified in two summary scores (physical component summary (PCS) and the mental component summery (MCS)).[8][9] To test the internal consistency of SF-36 in inhibitor patients, the Cronbach`s alpha was computed, with values greater than 0.70 considered satisfactory.[8] Descriptive statistics was also applied to describe HRQOL and health status measurement variables. Data were analyzed using Mann Whitney test.

The main demographic and clinical characteristics were shown in Table 1. Fifty percent of cases and 33% of controls had hepatitis C virus positive and 83% of cases and 33% of controls had a high degree of disability due to hemophilia. On average, the level of HRQOL shown by the sample of subjects was stable over time (data not shown). Our results showed that SF-36 gave lower scores in the dimensions more related to the physical components of health (physical functioning, role-physical and bodily pain) and higher scores in dimensions more related to the mental components (social functioning, role-emotional, mental health) both for inhibitors and non-inhibitors but scores in dimensions related to physical components in inhibitors are lower than in non-inhibitors (p-value=0.04) and the scores for physical dimensions showed more differences compared with this score for non-inhibitors. For dimensions equally related to both physical and mental health components, the general health score was relatively low, while the vitality score was higher. The global physical and mental measures mean scored were 36.9 and 50.2 respectively. Internal consistency of SF-36 was excellent, with Cronbach`s alpha values ranging from 0.8 to 0.9 for every dimensions of the questionnaire.

**Table 1:** Demographic and clinical characteristics of severe hemophiliacs with and without inhibitor.

**Variables**	**Value for inhibitors**	**Value for non-inhibitors**
Age (year)	25.5	25.6
Mean (±SD)	17.5	27.5
Median (min-max)	(11-32)	(10-33)
Weight (kg)	69	66
Mean (±SD)	67.5	69
Median (min-max)	(35-90)	(38-80)
Frequency of:		
HIV infection (%)	0	0
HBV infection (%)	0	0
HCV infection (%)	50	33.3
Disability: Patients with hemophilia-related disability (%)	83.3	33.3
Orthopedic functioning: Patients with choronic disability (%)	85.5	66.6

It appears that the expensive care of inhibitor patients led to an unsatisfactory level of HRQOL both in inhibitors and non-inhibitors and showed an important impairment of physical health perception and the mental domains, excluding social activities, did not show substantial differences. Substantial difference was found between inhibitors and non-inhibitors10 with regard to domains more related to the physical and mental components of health perception. Non-inhibitors had slightly higher values for vitality compare with inhibitors and higher for general health. Moreover, mental health perception, as summarized by the MCS score, was similar between these two groups. While the PCS score, pertaining to the physical component of health perception was higher in non-inhibitor patients.

Our results showed that the type of treatment used for inhibitors was not that much effective and these patients needed more care for physical status. Also this situation is similar for non-inhibitor patients. For those inhibitors by HCV or HBV, the related scores for physical dimension is less than the scores of those without HCV and HBV. The limitation of the study was small sample size and further study with larger sample size is suggested. In conclusion, inhibitor patients need more caring regarding optimal management.
